# Investigating retroviral envelope proteome plasticity

**DOI:** 10.1186/1742-4690-10-S1-P60

**Published:** 2013-09-19

**Authors:** Susanne Heider, Feliks Kochan, Sandra Kleinberger, Eva Sperl, Erik Reimhult, Christoph Metzner

**Affiliations:** 1 Institute of Virology, University of Veterinary Medicine Vienna, Vienna, Austria; 2 Institute of Biologically Inspired Materials, University of Natural Resources and Life Sciences Vienna, Vienna, Austria

## Background

We have recently demonstrated that under highly artificial conditions, retroviral envelopes can acquire new proteins carrying a post-translationally added glycosylphosphatidylinositol (GPI) anchor [1,2]. Subsequently, we are interested to see if retroviral particles may acquire novel protein functions post-exit under physiological conditions either using GPI-anchored or, eventually, other protein species. Therefore we are trying to assess how mutable the envelope proteome is in response to external stimuli. New functionalities acquired in such processes, i.e. protection from the complement system, may prove beneficial for the virus, especially in zoonotic infections.

## Materials and methods

Stably produced retro- and lenti-viral vectors based on HIV and MLV are used as viral model systems. Recombinantly produced GPI-anchored proteins carrying a 6xhis tag (monomeric green fluorescent protein - mGFP, the complement regulatory proteins CD55 and CD59 and Interleukin 2 - IL2) used as “insertion bait” molecules were purified via fast protein liquid chromatography (FPLC). Methods employed for analysis include immunoblotting, differential protein display and mass spectroscopy. Additionally, we are currently developing biophysical methodology for real-time label-free detection of changes at lipid bilayer surfaces, such as dual polarisation interferometry (DPI) or quartz crystal microbalances with dissipation monitoring (QCM-D), to follow insertion events.

## Results

Levels comparable to physiological levels of proteins suffice for insertion of GPI anchored protein. When impure preparations (i.e. presence of high protein background) were used, association of GPI anchored proteins is still possible. When GPI anchored mGFP, CD59, or IL2 were added to lenti-viral vector particles, the respective functions could be conferred to the vector particles.

## Conclusions

We have found that high protein backgrounds are permissive for insertion of low-abundance GPI anchored proteins and that protein functions are transferable with potential benefits for the virus (i.e. complement protection after transfer of CD55/CD59). Both findings support the hypothesis that retroviral particles may capture GPI anchored proteins from the surrounding media.
